# Machine learning and the role of the vaginal and fecal microbiome in miscarriage: a matched case-control study

**DOI:** 10.1038/s41522-026-00956-2

**Published:** 2026-03-13

**Authors:** Unnur Gudnadottir, Stefanie Prast-Nielsen, Nicole Wagner, Luisa W. Hugerth, Vilma Kuttainen Alderheim, Anusha T. Antony, Juan Du, Jorge Reis Guerreiro, Fredrik Boulund, Eva Wiberg-Itzel, Lars Engstrand, Ina Schuppe-Koistinen, Nele Brusselaers, Emma Fransson

**Affiliations:** 1 Department of Women’s and Children’s Health, Karolinska Institutet, Solna, Sweden; 2 Centre for Translational Microbiome Research, Department of Microbiology, Tumor and Cell Biology (MTC), Karolinska Institutet, Solna, Sweden; 3 Department of Clinical Neuroscience, Karolinska Institutet, Stockholm, Sweden; 4 Bioclinicum Lungforskningslab, Department of Medicine, Karolinska Institutet, Solna, Sweden; 5 Department of Medical Biochemistry and Microbiology, Science for Life Laboratory, Uppsala University, Uppsala, Sweden; 6 Department of Women’s and Children’s health, Uppsala University, Uppsala, Sweden; 7 Global Health Institute, Department of Family Medicine and Population Health, University of Antwerp, Antwerp, Belgium; 8 Department of Clinical Science and Education, Södersjukhuset, Stockholm Sweden; 9 Department of Public Health and Primary Care, Ghent University, Ghent, Belgium

**Keywords:** Microbiome, Microbiota

## Abstract

Miscarriage occurs in approximately 15% of all pregnancies, and recent studies have suggested a potential role of the microbiome. A nested case-control study from the Swedish Maternal Microbiome cohort was conducted, including 34 participants who sent at least one vaginal or fecal microbiome sample and questionnaire data before miscarrying (*n* = 34), and matched controls (*n* = 105 for regression models, *n* = 27 for machine learning models). Non-vaccine type HPV (aOR 3.95, 95%CI 1.04–15.06) and vaginal microbiome with community state type (CST) II (aOR 6.52, 95%CI 1.58–26.98) or CST-IVB (aOR 4.18, 95%CI 1.08–16.18) in early pregnancy were associated with an increased risk of miscarriage. Furthermore, we explored six machine learning algorithms using 70% of the cohort for training and 30% for testing, for the prediction of miscarriage using vaginal (AUROC 85%), fecal (AUROC 81%) and questionnaire (AUROC 82%) data separately and combined (AUROC 82%). Our results highlight the urgency of HPV screening and vaccine development for women’s reproductive health. Despite limitations, including a small number of miscarriage cases, our results indicate the potential for both vaginal and fecal microbiomes in the prediction of miscarriage.

## Introduction

Miscarriage, the spontaneous loss of a pregnancy before the fetus reaches the stage of viability, occurs in approximately 15% of all pregnancies^1–5^, and is usually defined as either early (before 12 completed gestational weeks) or late (gestational weeks 13-22). As one of the most frequent adverse events of pregnancy, it has a considerable impact on the physical and psychological well-being of the mother and relatives^5^.

In Sweden, nonviable pregnancies after week 22 are defined as intrauterine fetal demise/death (IUFD) or stillbirth^6–8^. Events such as miscarriage and stillbirth are complex with multifactorial etiology. Among the suggested risk factors for euploid (chromosomally “normal”) miscarriages are maternal infections (which may account for more than half of late miscarriages) and factors within the maternal microbiome^9,10^. Other risk factors include maternal age, obesity, multiple pregnancy, hypothyroidism, and immune system alterations^3,5,9–15^.

The human microbiome includes all microorganisms and their genetic material in a defined environment with specific conditions of a defined anatomical location, such as the oral cavity, intestines and vagina^16^. Pregnancy is a crucial event where significant microbial, metabolic, hormonal and immunological changes affect one another^17–19^, e.g. estrogen regulation by the gut microbiome^20^.

Recent studies have shown associations between the vaginal microbiome and miscarriage, where low Lactobacilli and high alpha diversity^21–25^ were associated with an increased risk of miscarriage^26^. Furthermore, *Lactobacillus iners* has been more commonly found in women with a history of miscarriage, compared to *L. crispatus*, the most common bacterial taxon in nulliparous women^27,28^. Low lactobacilli and high diversity in the vaginal microbiome have been linked to an increase in pro-inflammatory cytokines in women that experienced miscarriage^29^ and inflammatory mediators in women with repeated implantation failure and endometrial dysbiosis^29^.

Human papillomavirus (HPV) is the most common sexually transmitted infection, and current studies on the association of HPV and miscarriage have shown conflicting results^30^. It has been suggested that HPV may be more prevalent in pregnant women than non-pregnant^31^ and take longer to clear during pregnancy because of the altered immune response^30,32^.

Considering the gut microbiome, there are still relatively few studies performed regarding miscarriage, but an association with miscarriage has been suggested via lower diversity and increased levels of Th1/Th17 proinflammatory cytokines^33^. A decrease in *Bifidobacterium* and *Clostridiales* in the gut microbiome has also been associated with other adverse pregnancy outcomes, such as preterm birth^34^.

Most current studies on the topic focus on the vaginal microbiome. Here, we present data on the vaginal (including HPV sequencing data) and fecal microbiome using shotgun sequencing, as well as extensive questionnaire data, and evaluate multiple machine learning methods to create a prediction model for miscarriage. Our aim is to build a prediction model based on the most critical factors from all available data. Our secondary objective is to assess the association between the maternal microbiome (vaginal and fecal) in early pregnancy and the risk of miscarriage, considering background factors, medical and reproductive history, and prescription drug use, and a third aim is to compare microbiome profiles between those that had an intra-uterine fetal death (IUFD), participants that gave extreme or very preterm birth or had a history of recurrent pregnancy loss (RPL) with the miscarriage cases and controls.

## Results

In the cohort, 79 participants had confirmed miscarriage. Out of the 79 miscarriage cases, 34 sent in at least one microbiome sample (30 vaginal samples, 32 fecal samples), of whom 27 participants sent in both vaginal and fecal samples. Only those 27 that sent in both microbiome samples were included in the machine learning analysis.

In our cohort, miscarriages took place in weeks 10–20 (median 13, IQR 13–17), but for some participants, the gestational week of the miscarriage was unknown (*n* = 17). However, for all cases the miscarriage took place after answering the questionnaire and microbiome sampling, which was at the latest in pregnancy week 19. Miscarriage cases were matched with controls (98 vaginal samples, 105 fecal samples) that gave birth at term. Microbiome samples were taken during pregnancy weeks 10–19 for both cases and controls.

Additional comparison groups were participants that had previously experienced recurrent pregnancy loss but gave birth at term (*n* = 102, 102 vaginal samples and 102 fecal samples) or had spontaneous preterm birth or IUFD before gestational week 32 in the current pregnancy (*n* = 27, 27 vaginal samples and 26 fecal samples).

Most participants, both among miscarriage cases and matched controls, were 25–35 years old and average weight. No difference was found between HPV vaccination status of miscarriage cases and controls (18% for both).

Detailed descriptive features of the cohort are summarized in Table 1.

**Table 1:** Descriptive features of the cohort, stratified as miscarriage cases and matched controls, extreme/very preterm birth (PTB) or intrauterine fetal death (IUFD), and those with a history of recurrent pregnancy loss

	Control	Miscarriage	Extreme/very PTB* or IUFD**	History of recurrent pregnancy loss	Total
	(*N* = 105)	(*N* = 34)	(*N* = 27)	(*N* = 102)	(*N* = 268)
**Background factors**					
Age					
< 25	4 (4%)	1 (3%)	0 (0%)	4 (4%)	9 (3%)
25–35	52 (50%)	18 (53%)	19 (70%)	59 (58%)	148 (55%)
> 35	42 (40%)	11 (32%)	8 (30%)	39 (38%)	100 (37%)
BMI*** prior to pregnancy					
< 18.5 Underweight	5 (5%)	2 (6%)	0 (0%)	0 (0%)	7 (3%)
18.5–25 Normal weight	75 (71%)	21 (62%)	14 (52%)	61 (60%)	171 (64%)
> 25 Overweight	14 (13%)	4 (12%)	11 (41%)	33 (32%)	62 (23%)
Swedish born	83 (79%)	23 (68%)	25 (93%)	94 (92%)	225 (84%)
University education	89 (85%)	29 (85%)	23 (85%)	78 (76%)	219 (82%)
Family status					
Cohabiting	96 (91%)	30 (88%)	26 (96%)	101 (99%)	253 (94%)
Single	2 (2%)	0 (0%)	1 (4%)	0 (0%)	3 (1%)
Socioeconomic score					
High	26 (25%)	7 (21%)	10 (37%)	44 (43%)	87 (32%)
Low	72 (69%)	23 (68%)	17 (63%)	58 (57%)	170 (63%)
**Gynecological health**					
Ever had HPV****	18 (17%)	8 (24%)	6 (22%)	20 (20%)	52 (19%)
HPV vaccinated	19 (18%)	6 (18%)	2 (7%)	9 (9%)	36 (13%)
Ever had dysplasia	24 (23%)	6 (18%)	9 (33%)	29 (28%)	68 (25%)
VALENCIA^†^group					
CST-I	38 (39%)	9 (30%)	10 (37%)	27 (26%)	84 (31%)
CST-II	9 (9%)	7 (23%)	2 (7%)	5 (5%)	23 (9%)
CST-III	22 (23%)	6 (20%)	5 (19%)	38 (37%)	71 (26%)
CST-IVB	15 (16%)	7 (23%)	7 (26%)	18 (18%)	47 (18%)
CST-IVC	1 (1%)	1 (3%)	0 (0%)	1 (1%)	3 (1%)
CST-V	10 (10%)	0 (0%)	3 (11%)	6 (6%)	19 (7%)
No CST/Missing vaginal sample	10 (10%)	4 (12%)	0 (0%)	7 (7%)	21 (8%)
HPV					
Any HPV	8 (8%)	5 (15%)	6 (22%)	7 (7%)	26 (10%)
High risk HPV	6 (6%)	2 (6%)	3 (11%)	3 (3%)	14 (5%)
Low risk HPV	1 (1%)	1 (3%)	1 (4%)	1 (1%)	4 (1%)
Vaccine type HPV	2 (2%)	0 (0%)	1 (4%)	1 (1%)	4 (1%)
Non-vaccine type HPV	6 (6%)	5 (15%)	5 (19%)	6 (6%)	22 (8%)
Missing vaginal sample	7 (7%)	4 (12%)	0 (0%)	0 (0%)	11 (4.1%)
**General health**					
Smoking	0 (0%)	0 (0%)	0 (0%)	3 (3%)	3 (1%)
Snuff / smokeless tobacco	0 (0%)	0 (0%)	1 (4%)	0 (0%)	1 (0%)
Good self-health estimation	94 (90%)	29 (85%)	25 (93%)	89 (87%)	237 (88%)
Health-seeking behavior	90 (86%)	30 (88%)	21 (78%)	90 (88%)	231 (86%)
Daily fiber	88 (84%)	29 (85%)	26 (96%)	87 (85%)	230 (86%)
Eating disorder	9 (9%)	4 (12%)	2 (7%)	9 (9%)	24 (9%)
**Pregnancy characteristics**					
Primiparous	45 (43%)	11 (32%)	17 (63%)	23 (23%)	96 (36%)
Alcohol during pregnancy	9 (9%)	2 (6%)	2 (7%)	6 (6%)	19 (7%)
Regular menstruation	93 (89%)	26 (76%)	22 (81%)	84 (82%)	225 (84%)
High stress early pregnancy	29 (28%)	7 (21%)	11 (41%)	34 (33%)	81 (30%)
EPDS‡ depression score early pregnancy					
Mean (SD)	5.5 ( ± 3.7)	5.5 ( ± 4.1)	6.5 ( ± 5.4)	7.7 ( ± 5.2)	6.5 ( ± 4.7)
≥ 12	8 (8.5%)	7 (12.9%)	6 (25%)	21 (22.6%)	10 (16.1%)
Natural conception	92 (88%)	27 (79%)	23 (85%)	95 (93%)	237 (88%)
Time to pregnancy					
0–6	76 (72%)	20 (59%)	19 (70%)	51 (50%)	166 (62%)
6–12	7 (7%)	3 (9%)	2 (7%)	9 (9%)	21 (8%)
> 12	15 (14%)	7 (21%)	6 (22%)	42 (41%)	70 (26%)
Any pregnancy problems	11 (10%)	4 (12%)	8 (30%)	19 (19%)	42 (16%)
PUQE^††^ score					
Mild	73 (70%)	25 (74%)	21 (78%)	75 (74%)	194 (72%)
Moderate	23 (22%)	4 (12%)	6 (22%)	27 (26%)	60 (22%)
Severe	2 (2%)	1 (3%)	0 (0%)	0 (0%)	3 (1%)
Bristol stool scale rank					
Fast transit	29 (28%)	10 (29%)	6 (22%)	23 (23%)	68 (25%)
Normal transit	25 (24%)	7 (21%)	8 (30%)	15 (15%)	55 (21%)
Slow transit	28 (27%)	11 (32%)	6 (22%)	31 (31%)	76 (28%)
Various transit	23 (22%)	5 (15%)	7 (26%)	32 (32%)	67 (25%)
**Drug use during pregnancy**					
Any drugs	37 (35%)	10 (29%)	11 (41%)	58 (57%)	116 (43%)
Multiple drugs	35 (33%)	8 (24%)	9 (33%)	53 (52%)	105 (39%)
Allergy or antihistamine medication	13 (12%)	3 (9%)	0 (0%)	18 (18%)	34 (13%)
Neurological medication	6 (6%)	3 (9%)	2 (7%)	12 (12%)	23 (9%)
Hematological medication	4 (4%)	2 (6%)	3 (11%)	14 (14%)	23 (9%)
Blood pressure medication	0 (0%)	2 (6%)	1 (4%)	5 (5%)	8 (3%)
Antibiotics	4 (4%)	2 (6%)	2 (7%)	5 (5%)	13 (5%)

^*^Preterm birth, **Intrauterine fetal death, ***Body mass index, ****Human Papillomavirus

†Vaginal community state type nearest centroid classifier, ‡Edinburgh Postnatal Depression Scale, ††Pregnancy-Unique Quantification of Emesis score,

No descriptive characteristics were statistically different when comparing miscarriage cases and controls (*p* < 0.05) using Chi-square tests. However, some variables of interest showed non-significant differences. Women who had suffered miscarriage were more often born outside of Sweden (32% vs. 21%), were multiparous (68% vs. 57%), had irregular menstruation (24% vs. 11%) and had undergone assisted conception (21% vs. 12%) compared to matched controls. Miscarriage cases also needed extended time to conceive, with 23% vs. 14% needing >12 months to conceive. Miscarriage cases more commonly reported ever having HPV (24% vs. 17%), and vaginal microbiome data showed they more often had any HPV (15% vs. 8%) and non-vaccine type HPV (12% vs. 7%) at the time of sampling.

### Vaginal microbiome

**Fig. 1:**
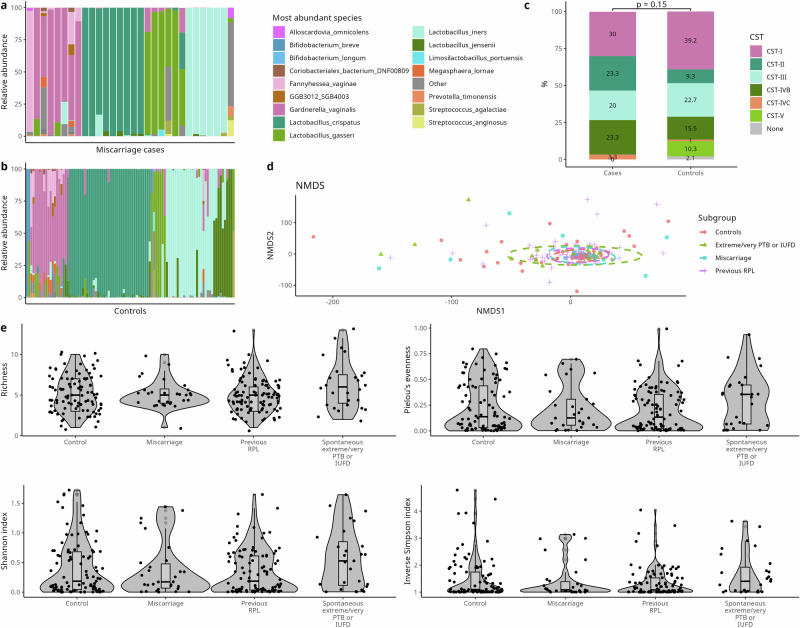
Vaginal microbiome data of the cohort. Relative abundance of the 20 most abundant species of miscarriage cases (**a**) and matched controls (**b**). **c** Cases and controls are grouped into VALENCIA CSTs. **d** NMDS plot using Aitchison distance for cases (blue), controls (pink), those with a history of RPL (purple) and those with extreme/very spontaneous PTB or IUFD (green) with *p*-values from PERMANOVA analysis. **e** Violin plots showing Richness, Pielou’s evenness, Shannon index and Inverse Simpson index of the four subgroups.

When comparing the distribution of vaginal microbiota CSTs in miscarriage cases to controls (matched 1:3) (Fig. 1a, b), cases had a lower prevalence of CST-I (*L. crispatus)* (30.0% vs. 39.2%) and CST-V (*L. jensenii)* (0% vs. 10.3%) and higher prevalence of CST-IVB (23.3% vs. 15.5%). These differences in prevalence between cases and controls were not significant in a Chi-squared test when considered as one variable (*p* = 0.15) (Fig. 1c), or when each CST was investigated as a separate variable (CST-I *p* = 0.40, CST-II *p* = 0.06, CST-III *p* = 1, CST-IVB *p* = 0.41, CST-IVC *p* = 0.42, CST-V *p* = 0.12).

No significant difference in beta diversity was found between miscarriage cases and controls (Fig. 1d), nor between cases and those that had a history of recurrent pregnancy loss or experienced spontaneous extreme or very preterm birth.

PERMANOVA univariable analysis showed a significant association between the vaginal microbiome and low-risk HPV (*p* = 0.02, R^2^ = 0.03) and vaccine type HPV (9 valent) (*p* = 0.002, R^2^ = 0.05) (Table S3).

A Wilcoxon Rank-Sum test showed no difference between miscarriage cases and controls regarding Shannon index (*p* = 0.1), Inverse Simpson (*p* = 0.98), Pielou’s evenness (*p* = 0.98) or Richness (*p* = 0.98) for vaginal data (Fig. 1e). An ANOVA test showed no difference between the four groups for Shannon index (*p* = 0.14), Inverse Simpson (*p* = 0.16), Pielou’s evenness (*p* = 0.21) or Richness (*p* = 0.12).

A Wilcoxon Rank-Sum test showed no difference for the same metrics when comparing those with a history of RPL to miscarriage cases and controls (separately) or those with spontaneous early/very PTB or IUFD to cases and controls (separately) (Fig. 1e).

### Fecal microbiome

**Fig. 2:**
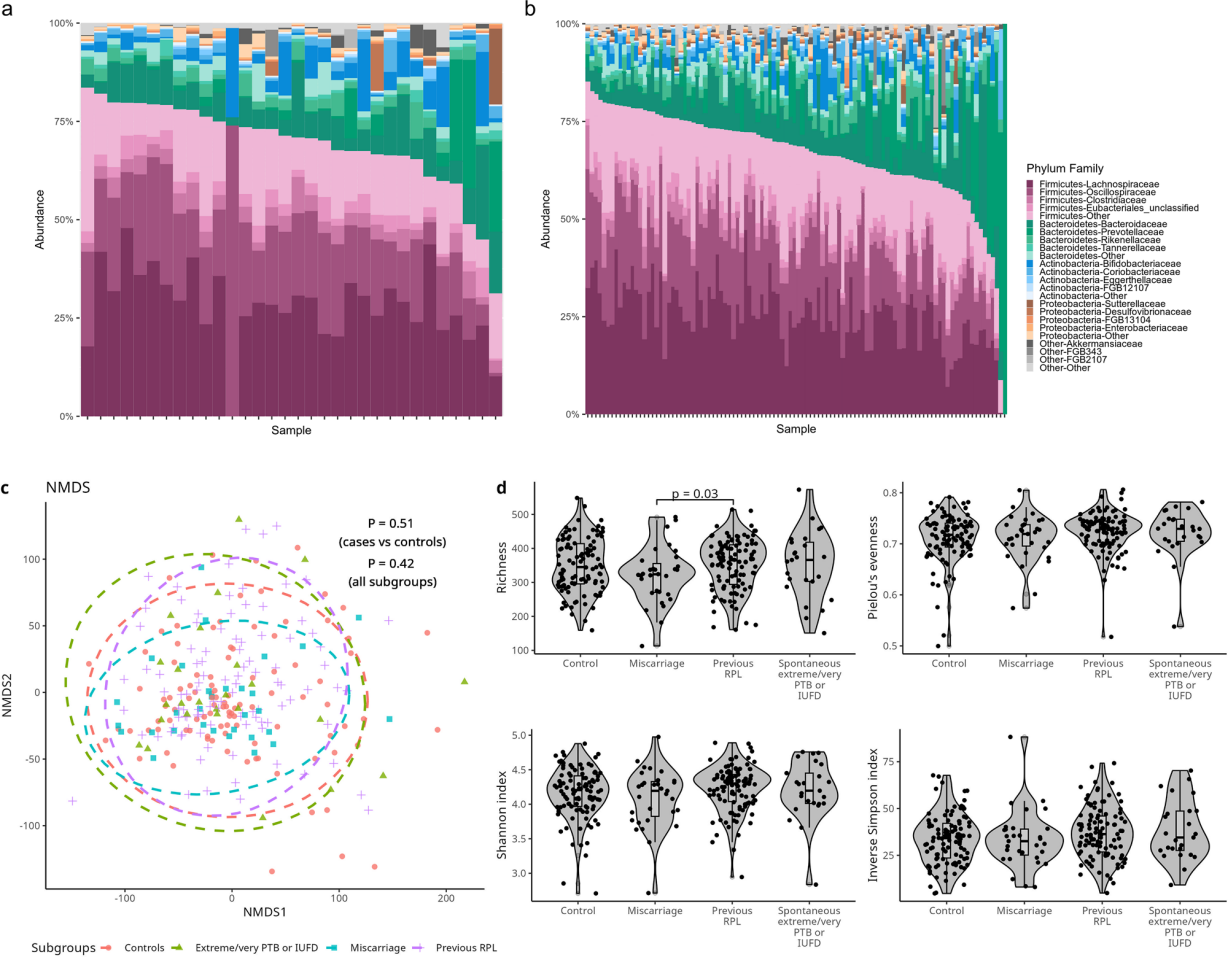
Fecal microbiome data of the cohort. Relative abundance of phyla and family of the miscarriage cases (**a**) and matched controls (**b**). **c** NMDS plot using Aitchison distance for cases (blue), controls (pink), those with a history of RPL (purple) and those with extreme/very spontaneous PTB or IUFD (green) and p values from PERMANOVA analysis. **d** Violin plots showing Richness, Pielou’s evenness, Shannon index and Inverse Simpson index of the four subgroups.

Relative abundance was plotted in a stacked bar plot showing phylum and family, with Firmicutes, Bacteroidetes, and Actinobacteria being most prevalent (Fig. 2a, b).

The NMDS plot using Aitchison distances showed no significant difference between cases and controls as well as between subgroups (Fig. 2c).

PERMANOVA univariable analysis showed a significant association between the fecal microbiome and age (*p* = 0.03, R^2^ = 0.02), country of birth (*p* = 0.04, R^2^ = 0.01), education (*p* = 0.05, R^2^ = 0.01), parity (*p* = 0.002, R^2^ = 0.01), and using hematological (including aspirin and blood thinners) (*p* = 0.05, R^2^ = 0.01) or blood pressure medication in early pregnancy (*p* = 0.05, R^2^ = 0.01) (Table S3).

A Wilcoxon Rank-Sum test showed no difference for miscarriage cases and controls considering Shannon index (*p* = 0.44), Inverse Simpson (*p* = 0.72), Pielou’s evenness (*p* = 0.84) or Richness (*p* = 0.14). An ANOVA test showed no difference between the four groups for Shannon (*p* = 0.25), Inverse Simpson (*p* = 0.18), Pielou’s evenness (*p* = 0.21) or Richness (*p* = 0.29). However, a Wilcoxon Rank-Sum test showed a difference in richness when only comparing those with a history of RPL to miscarriage cases (Shannon *p* = 0.07, Inverse Simpson *p* = 0.12, Pielou’s evenness *p* = 0.15, Richness *p* = 0.03). No significant difference was observed when comparing those with spontaneous very/extreme PTB or IUFD to miscarriage cases and controls (Fig. 2d).

### Logistic regression models

In the univariable analysis having any HPV, non-vaccine type HPV, vaginal microbiome CST-II, irregular menstruation and Inverse Simpson index for the fecal microbiome had *p* < 0.20 and were therefore included in the multivariable model (Table S1).

A multivariable model (adjusted for having non-vaccine type HPV, CSI-II, irregular menstruation and Inverse Simpson index of fecal microbiome) showed that having CST-II (aOR 6.52, 95%CI 1.58–26.98) or CST-IVB (aOR 4.18, 95%CI 1.08–16.18) or non-vaccine type HPV (aOR 3.95, 95%CI 1.04–15.06) were associated with an increased risk of miscarriage. On the other hand, having regular menstruation was associated with a decreased risk of miscarriage (aOR 0.16, 95%CI 0.03–0.78) (Table S1).

Assuming causality, the population attributable fraction of miscarriage for CST-II (with CST-I as reference) was 24.6%, 17.4% for CST-IVB (with CST-I as reference) and 7.3% for non-vaccine type HPV (with no HPV as reference).

### Machine learning models for the prediction of miscarriages

When investigating the complete four different datasets, which were all balanced with cases and controls 1:1, different algorithms performed best (highest average AUROC over the five test sets) depending on datatype (Table 2, Table S4).

**Table 2:** Most important variables in the best machine learning model for each dataset with AUROC scores and their 95% confidence intervals (Cis) for the five test sets using both the full models and restricted models after feature selection

Dataset	Accuracy full model	Average AUROC*	Algorithm of best full model (highest AUROC)	AUROC after feature selection	Most important variables (from most important to least)
Fecal microbiome (1858 variables, including diversity metrics)	43–76%	56–79%	Ranger Random Forest (79%, 95%CI 76–82%)	81% (95%CI 74–87%) (5 variables)	GGB58485 SGB80143 (class Clostridia), GGB9759 SGB15370 (class Clostridia), GGB9786 SGB63164 (class Clostridia), Anaerococcus SGB15407 (class Clostridia), GGB9778 SGB15398 (class Clostridia)
Vaginal microbiome (211 variables, including diversity metrics and CSTs)	30–64%	54–85%	svm (85%, 95%CI 83–87%)	78% (95%CI 74–82%) (70 variables)	*Top 20 out of 70:* Corynebacterium aurimucosum, Prevotella colorans, Non-vaccine-type HPV, Aerococcus christensenii, Other vaginal bacteria, Streptococcus SGB3665, Lactobacillus jensenii, Staphylococcus hominis, Corynebacterium sp HMSC08D02, any HPV, Megasphaera lornae, Lactobacillus crispatus, Anaerococcus hydrogenalis, Prevotella bivia, Inverse Simpson, GGB38873 SGB47522, Schaalia turicensis, Streptococcus urinalis, Atopobium deltae, GGB753 SGB989
Questionnaire data (29 variables)	45–60%	47–77%	svm (77%, 95%CI 71–83%)	82% (95%CI 76–87%) (20 variables)	Bristol rating early pregnancy, HPV vaccination, parity, multiple drugs in early pregnancy, daily fiber, EPDS score, regular menstruation, age, education, antibiotics during early pregnancy, neurological medication early pregnancy, any drugs early pregnancy, socio-economic score, healthy diet, natural conception, family status, good self-estimation of health, prior mental diseases, PUQE score, blood pressure medication during early pregnancy
All combined (95 variables: top 5 fecal, top 70 vaginal and top 20 questionnaire data)	58–73%	62–82%	Ranger Random Forest (82%, 95%CI 76–89%)	81% (95%CI 75–87%) (5 variables) and 80% (95%CI 74–86%) (30 variables)	*From fecal microbiome:* GGB58485 SGB80143, GGB9759 SGB15370, GGB9786 SGB63164, Anaerococcus SGB15407, GGB9778 SGB15398 *From vaginal microbiome:* Gardnerella vaginalis Arthrobacter sp HMSC06H05, Corynebacteriu sp HMSC08D02, Corynebacterium tuscaniense, Coriobacteriales bacterium DNF00809, Mobiluncus curtisii, Slackia exigua, Corynebacterium kroppenstedtii, Schaalia turicensis, Bacteroidales_bacterium_KA00251, Actinomyces sp HMSC065F12, Actinomyces urogenitalis, Actinotignum sanguinis *From questionnaire:* Parity, pregnancy problems in early pregnancy, high stress, blood pressure medication in early pregnancy, age, BMI, family status, good self-estimation of health, PUQE score, antibiotics during pregnancy, hematological drugs during pregnancy

^*^AUROC area under receiver operating characteristic curve, **HPV human papilloma virus, ***PUQE pregnancy-unique quantification of emesis score, ****BMI body mass index.

**Fig. 3:**
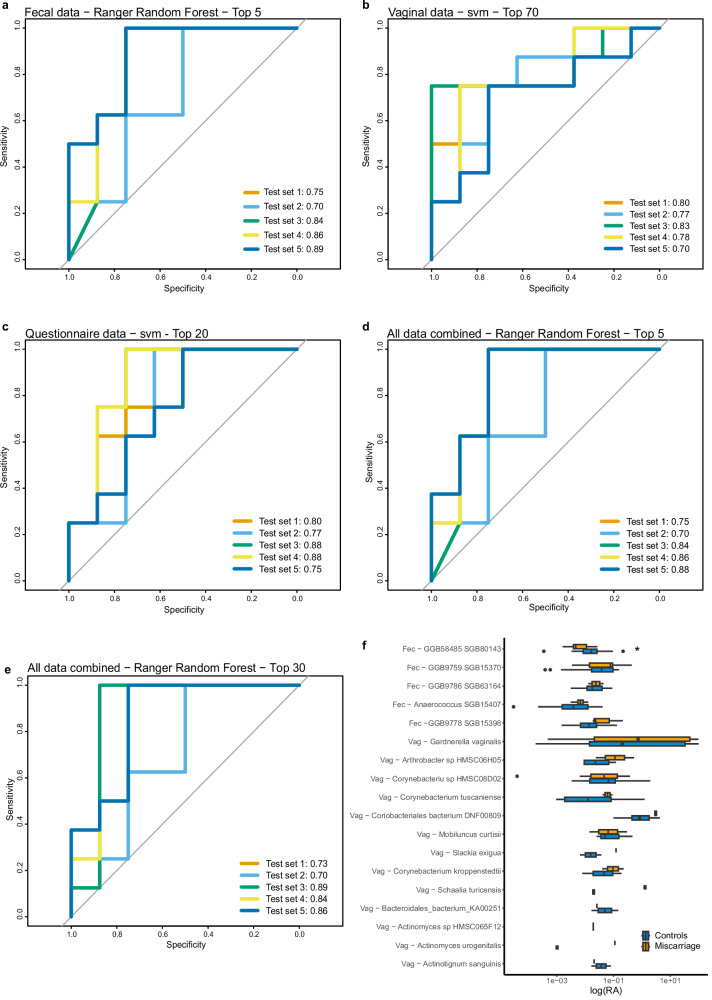
Machine learning results for the different data sets. AUROC values for the five test sets using the best-performing model after feature selection for (**a**) fecal (Ranger Random Forest), (**b**) vaginal (svm), (**c**) questionnaire data (svm), and (**d**, **e**) all combined (Ranger Random Forest), and (**f**) the logarithm of the relative abundance of the fecal (fec) and vaginal (vag) species included in the top 30 combined Ranger Random Forest model (**e**), with an asterisk (*) indicating significant differential abundance (*p* < 0.05) in Wilcoxon Rank-Sum test.

For the full fecal dataset, Ranger Random Forest performed best (AUROC 79%), which increased to 81% (95%CI 74–87%) after feature selection (top 5 variables) (Fig. 3a).

When testing the full vaginal dataset, svm performed best (AUROC 85%). In feature selection, when testing the top 20, 10 or 5 most important variables they all performed worse than the full model. The top 80, 70, 60, 50, 40 and 30 variables were therefore also tested. Out of all the denoised models, the one with the top 70 variables performed best (AUROC 78%, 95%CI 74–82%), but still not as well as the full model (Fig. 3b).

For the full questionnaire data (Table S3), svm performed best (AUROC 77%), and achieved an AUROC of 82% (95%CI 76–87%) after feature selection (top 20 variables) (Fig. 3c).

When combining the variables ranked most important in these three datasets (top 20 questionnaire, top 5 fecal and top 70 vaginal), the best model was achieved using the Ranger Random Forest algorithm (AUROC 82%, 95%CI 76–89%) (Table 2). After feature selection, the AUROC remained similar, at 81% (95%CI 75–87%) (top 5 variables) (Fig. 3d). As all the top 5 variables were derived from the fecal microbiome, the top 30 variables are also presented (Fig. 3e), as that model had the similar AUROC (80%, 95%CI 74-86%) but used data from all three sources (Fig. 3f). This model had Brier score of 0.31, sensitivity of 82%, specificity of 89%, positive prediction value of 89% and negative prediction value of 83%. Stability of important features across folds for this model was on average 0.65 (Spearman correlation), indicating a moderate stability.

The accuracy of the top five model over the five test sets was 74%, and 70% for the top 30 model.

In the combined model (with 30 variables), the five fecal species included were ranked as the most important (all belonging to class Clostridia), followed by a combination of vaginal variables (13 variables) and questionnaire variables (12 variables) (Table 2, Fig. 3f, Figure S1). When investigating the relative abundance of the included species, one of them (fecal) was found to be differentially abundant in a Wilcoxon rank-sum test (Fig. 3f).

## Discussion

In this study, we present a machine learning model to predict miscarriage, with high specificity and AUROC value. We assessed four different datasets (questionnaire data, vaginal microbiome data, fecal microbiome data and all data types combined) using six different machine-learning algorithms. We found that accuracy and AUROC values varied depending on the method used, emphasizing the importance of testing multiple machine-learning models in microbiome analyses. By using state-of-the-art machine learning methods, we were able to identify specific bacteria with predictive potential for miscarriage, even though no differences were observed in alpha and beta diversity of the same cohort. This is partially due to machine learning models recognizing patterns and giving different species different rankings of importance when predicting the outcome^35^.

All models performed similarly with regards to AUROC, which could be beneficial clinically to use whichever data is most readily available. We also found that certain drug groups (blood pressure medication, hematological medication, and antibiotics) were used to classify cases and controls, which could also be proxies for underlying conditions affecting the risk of miscarriage.

Furthermore, in our multivariable logistic regression model we found that being positive for any non-vaccine HPV or having a vaginal microbiome CST-II or CST-IVB were associated with a four-to-six-fold increased risk in miscarriage, compared to those with CST-I. HPV has previously been associated with a dysbiotic and high diversity vaginal microbiome^36^, and either HPV could cause a shift in the microbiome, or a higher diversity microbiome could be more susceptible to HPV infection. On the other hand, having regular menstruation was associated with a decreased risk of miscarriage.

In addition to its significance in our logistic regression model, non-vaccine type HPV was also incorporated in the best vaginal machine learning model. This association has been reported in some previous studies, while others disagree^10,30,37^. The difference in results could depend on the timepoint of miscarriage (early or late), type of miscarriage (silent or not, chromosomal disfunction or not – information we did not have for the present study), or other risk factors found in the different study populations. In this study, samples were on average taken in pregnancy week 13, while most studies on HPV and miscarriage have samples from the first trimester (up to week 13)^38–40^. HPV infections are the most prevalent genital infections in women. While some high-risk types can be avoided with vaccination, it may be more challenging to prevent non-vaccine types. HPV prevalence in Sweden is very high, with a recent study finding HPV in 67.5% of women attending a youth clinic and 34.1% of those attending routine cervical screening^41^. Furthermore, in a PERMANOVA analysis of the vaginal microbiome, including HPV data, an association with beta diversity in the microbiome was seen for low-risk HPV, non-vaccine type HPV and parity. Being HPV-positive has previously been associated with increased diversity in the vaginal microbiome^41^. Currently, HPV is mainly studied concerning cervical cancer, but our results highlight the need to include it in studies on fertility and pregnancy.

When evaluating the association of the fecal microbiome to selected questionnaire variables, some expected features were found to be significant, such as age^42^, country of birth^43^, and parity^44^. An association was also found with hematological medication and blood pressure medication, both of which were incorporated in the best combined machine learning model.

The subgroup analyses of the fecal microbiome revealed a significantly higher richness for individuals with a history of RPL compared to the miscarriage cases. Current literature on the gut microbiome of RPL patients is scarce, with one study reporting higher diversity in their gut microbiome compared to controls^45^, and another study that shows lower fecal diversity in RPL patients^33^.

A major strength of this study is the combination of multiple data types, as most studies on miscarriage only include vaginal microbiome data. As this is a nested case-control study, it allowed us to collect pre-miscarriage samples, which can be difficult to acquire but are crucial for this research. Additionally, top-of-the-line analysis methods such as shotgun sequencing for microbiome analysis, standardized extensive questionnaires, and machine learning approaches add to the study’s strength. Furthermore, as both controls and miscarriage cases answer the questionnaire in early pregnancy (before the miscarriage happens), we can minimize recall bias.

This study also has limitations. The number of miscarriage cases limits our analysis, as can be seen in the size of the confidence intervals in the multivariable logistic regression. It may cause variables associated with miscarriage to be non-significant in our analysis. Even though the confidence intervals are broad, the results regarding HPV and the risk of miscarriage indicate that this relationship should be further studied in larger cohorts. Furthermore, due to our low power, some variables we found to be non-significant but close to significance in our logistic regression model and PERMANOVA analysis could be significant if assessed in larger cohorts. Additionally, potential misclassification is caused by a lack of information about whether the miscarriages were aneuploid or had any chromosomal defects and the gestational week of the miscarriage was sometimes missing. Since all miscarriages included in this study took place at the end of the first trimester or the beginning of the second trimester ( > week 10 of gestation), chromosomal abnormalities are less likely to be the leading cause^46^. Nevertheless, the information on the week of miscarriage can be difficult to obtain since some may have been silent and happened before microbiome sampling but was only discovered later in the pregnancy. The interval between miscarriage and sampling in this study varies by some weeks and would be a valuable factor to include in future studies. However, importantly, as far as we know, the sampling was performed before the miscarriage was reported by the participant. This ensures that the microbiome is the exposure and the miscarriage is the outcome and not vice-versa. Another limitation is self-selection bias, which often accompanies volunteer cohorts, as previously discussed in our cohort description^47^, and self-reporting since data on miscarriage were obtained from the participants themselves and not from medical records. The SweMaMi cohort includes mostly participants with a high education level and many participants with a history of pregnancy complications such as recurrent pregnancy loss. Therefore, validating our results on larger and more diverse cohorts is needed before clinical implementation of a prediction model.

Even though clinical implementation of microbiome data is unlikely, our results on HPV association and the models, including easy-to-access background data, could be utilized after further validation and testing. The use of the microbiome clinically includes using it as a screening tool or as a probiotic target for treatments. Also, as HPV is already recognized by the public concerning cervical cancer, emphasizing the importance of vaccinations, safe sex with condoms (when not trying to conceive), and regular screening could help increase awareness and decrease the number of HPV-positive individuals. Although the top-performing model achieved an accuracy of 74%, which could be of clinical value for couples attempting to conceive, the application of machine learning methods to this type of data may be too labor-intensive for routine clinical implementation and evaluation. Nevertheless, such approaches may help guide future research by identifying specific bacterial taxa warranting further investigation, such as the class Clostridia, which was consistently ranked as the most important group among all the fecal species in our models. Given that logistic regression models are be easier to perform and interpret, they may be more suitable for clinical risk assessments in early pregnancy in similar populations, before complex models are in place. However, prior to any clinical implementation, these findings must be validated in larger and more diverse cohorts, as the present study is based on a small and homogeneous sample.

To conclude, we found non-vaccine type HPV infection and a vaginal microbiome dominated by less common Lactobacilli to be associated with an increased risk of miscarriage. Additionally, women who had a history of RPL but delivered at term in their current pregnancy had significantly higher richness in their fecal microbiome compared to miscarriage cases. Furthermore, clinically available background variables, fecal and vaginal microbiome data combined showed the potential use in predicting late miscarriage. Early prediction of women at risk for miscarriage may be clinically relevant for individuals going through in vitro fertilization or those who have suffered recurrent pregnancy loss. Increased knowledge of the role of the microbiome in miscarriage might provide new preventive treatment options in the future to women at risk.

## Methods

The Swedish Maternal Microbiome (SweMaMi) cohort included pregnant participants in Sweden from 2017 to 2021, as previously described in ref. ^47^. Briefly, samples for microbiome analysis (self-sampled vaginal and fecal samples) and standardized questionnaires were collected at three timepoints: I) Gestational week 10–20, II) Gestational week 28–32 and III) five weeks postpartum. Only samples from the first timepoint were used in this study. Furthermore, a linkage between high-quality national Swedish registries and participant data was established.

The SweMaMi cohort study was approved by the Regional Ethics Committee in Stockholm on 29 July 2017 (2017/3:7), and the National Ethics Committee approved an amendment on 13 May 2020 (Dnr 2020-01629). All study procedures were performed according to international guidelines for Good Clinical Practices (GCP) and the Helsinki Declaration for ethical principles for medical research.

### Study design

In this nested case-control study all women who self-reported a miscarriage after collection of the first microbiome sample within the cohort were included. A participant was categorized as having had a miscarriage if they reported either a miscarriage or a missed abortion before 22 completed pregnancy weeks.

Each case of miscarriage was matched 1:3 with participants who delivered at term (>39^+^0 gestational weeks) without a history of recurrent pregnancy loss (RPL), polycystic ovary syndrome (PCOS) or endometriosis, using the nearest neighbor model in the R package MatchIt^48^. Each control was matched for maternal age (continuous), body mass index (BMI, as three groups: underweight, average weight, and overweight or obese), gestational week at the time of microbiome sampling (continuous), and period of sampling (pre- or post-Covid-19, defined as before or after March 1^st^, 2020).

Additionally, cases of intrauterine fetal demise (IUFD), spontaneous extremely/very preterm birth (before 32 completed weeks of gestation) and those with a history of recurrent miscarriage (but who gave birth at term) were compared to those with miscarriage in subgroup analyses. In this study, we use the definition of RPL as having had three or more pregnancy losses, either consecutive or not^5^.

### Data collection

For this study, only microbiome samples and questionnaires from the first timepoint were used for cases and controls, as well as registry data on gestational length at birth for the controls.

A detailed description of the SweMaMi project, extraction and sequencing can be found in the published cohort profile^47^. Briefly, web questionnaires were answered in early pregnancy (before week 20), and microbiome samples were then self-collected by participants between gestational weeks 10–20. Fecal and vaginal samples were placed in DNA/RNA shield (Zymo Research, California, USA) and mailed to the research team. Samples were then stored at −80 °C before bead beating in a FastPrep 24 5 G and DNA extraction with the ZymoBIOMICS 96 MagBead DNA protocol. Shotgun sequencing was performed using an MGI T7 sequencer with PE150 reads. Sequencing data trimming was performed with fastp (default settings, version 0.23.2)^49^, host DNA removal by annotating with Kraken2 (version 2.1.2)^50^ against a database with only the GRCh38 human genome and taxonomy annotation with metaphlan4.0^51^, within the StaG-mwc framework (version 0.5.1)^52^.

### Data analysis

Both vaginal and fecal microbiome samples were filtered by excluding any species that did not appear in at least two samples and removing any species which had a relative abundance below 0.01% in all samples combined to remove possible contamination and false positives. This left 199 vaginal species (out of 1227) and 1865 fecal species (out of 3189). Furthermore, fecal samples with richness (number of species per sample) <50 were excluded from analysis (2 controls, 1 case, 1 subgroup analysis case).

For relative abundance in vaginal samples, the top 19 species in all samples were selected, and less common species were grouped as “Other” to give a total of 20 variables. Samples were categorized into community state types (CSTs) using the nearest centroid classification VALENCIA^53^. The difference in community-type prevalence between cases and controls was studied using the Chi-square test. The five most abundant phyla were selected to visualize the relative abundance of fecal samples, and the remaining phyla were grouped as “Other”. The occurrence of the Vaginal Human Papilloma Virus (HPV) in the vaginal sequencing data was quantified using HPViewer (Hao, Yang et al. 2018) and used as a binary variable (presence/absence) for high-risk HPV (16, 18, 31, 33, 34, 35, 39, 45, 51, 52, 56, 58, 59, 66, 68, and 70), low-risk HPV (6, 11, 42, 43, and 44), vaccine type (6, 11, 16, 18, 31, 33, 45, 52 and 58) and non-vaccine type^54,55^ after filtering away all types with < 5 reads.

The relative abundance of fecal microbiome data was visualized using the R packages microshades (v1.13)^56^ and phyloseq (v1.46.0)^57^, showing Phylum and Family for each sample.

Alpha-diversity was estimated using the Shannon index, inverse Simpson, Pielou’s evenness and observed species richness calculated with the R package vegan (v2.6-6.1)^58^. The diversity was compared using ANOVA for all groups and Wilcoxon rank-sum tests for (1) Miscarriage cases and controls, (2) Miscarriage cases and those with previous RPL, (3) Miscarriage cases and those with spontaneous extreme or very preterm birth or suffered IUFD.

For beta-diversity comparison, samples were center log ratio (CLR) transformed after adding 10% of the smallest relative abundance value to all values (to remove zeros). NMDS using Aitchison distance was used to compare cases, controls, those with RPL history, and those with spontaneous preterm birth before gestational week 32 or IUFD in the current pregnancy.

Multivariable logistic regression assessed the association between microbiome variables and miscarriage. For exposure, the diversity variable for vaginal and fecal microbiome data, which performed best in the univariable analysis, was included in the multivariable model. The following maternal risk factors for miscarriage were considered as confounders or moderators: (1) Background factors; age (as three categories: < 25, 25−35, >35), body mass index (BMI, as categories: underweight (<18.5 kg/m^2^), average weight (18.5–24.9 kg/m^2^), overweight and obese (>25 kg/m^2^)), birth country (Sweden or other), education (University level or less), family status (single or cohabiting), socioeconomic status (high if university education, cohabiting and working full time), (2) General health; smoking, snuff/smokeless tobacco, self-rated health (good vs average or bad), health seeking behavior (yes if never smoking, never snus/smokeless tobacco, not drinking alcohol during pregnancy, alcohol prior to pregnancy 2−4 times a month or less, self-rated health good or very good, sugary drinks once a week or less, fruits daily or often/week), diet (daily fiber or less frequent), diagnosed eating disorder, (3) Pregnancy characteristics; parity, alcohol use during pregnancy (the Alcohol Use Disorder Identification Test^59^), high stress (highest quartile in the Perceived Stress Scale (PSS-4)^60^), depressive symptoms score (the Edinburgh Postnatal Depression Scale (EPDS), continuous^61^ and with cut-off ≤ 12), method of conception (assisted or not), any pregnancy problems in current pregnancy (gestational diabetes, thyroid disease, high blood pressure, hyperemesis gravidarum, depression, vaginal bleeding, heartburn, symphysis pubis dysfunction or other), pregnancy-unique quantification of emesis (PUQE) score (grouped as mild, moderate or severe^62^), Bristol stool scale during early pregnancy (fast, normal, slow or various transit) 4) Drug use during pregnancy, 5) Gynecological health; regular menstruation, ever having had HPV, ever having had dysplasia, HPV vaccination status. Potential confounders/moderators that showed *p* < 0.20 in the univariable logistic regression analysis were included in a multivariable logistic regression model of microbiome variables and miscarriage. Results were presented as adjusted odds ratio (aOR) and 95% confidence intervals (CI). Population-attributable fractions were calculated for significant variables.

Background questionnaire variables were compared between cases and controls using Chi-square tests, where *p* < 0.05 was considered significant.

To investigate the association between the microbiome, miscarriage and questionnaire variables, PERMANOVA analysis was performed using Aitchison distance in the adonis2 function from the vegan package (v2.6-6.1)^58^.

### Machine learning prediction modelling

For the machine learning models, matched controls were randomly chosen to maintain a 1:1 ratio of cases and controls. To assess the predictive capacity of the microbiome data and questionnaire variables on the risk of miscarriage, the following machine learning methods (Support Vector Machines with Radial Basis Function Kernel (svmRadial)), Elastic Net, classic Random Forest, Ranger Random Forest, Neural Networks with feature extraction, and k-Nearest Neighbor were used within the caret (v6.0-94) package^63^. Models were built using (1) questionnaire data, (2) vaginal microbiome (CLR transformed, including Shannon index, reverse Simpson and richness and HPV results) and (3) fecal microbiome data (CLR transformed, including Shannon index, reverse Simpson and richness) or (4) best performing variables of all data types combined. Data was split into training (70%) and testing (30%) datasets using fixed splits^63^. Cross-validation was performed using leave-one-out and grid search to find the optimal model hyperparameters within the folds^63^. Testing was repeated five times using the same cases, where controls were randomly resampled from the control pool for each testing run to create five test sets. Feature selection was performed on each dataset’s best models (highest AUC value and/or accuracy), selecting the most important 20, 10 or 5 features where feasible and training a new model including only the selected variables. The default 0.5 probability threshold was used for all models. The machine learning code is available on GitHub (see code availability statement).

Results were reported as accuracy (%) of the models on the test datasets, receiver operating characteristic (ROC) curves with area under the ROC curve (AUROC with 95% confidence intervals (CIs), values (mean over the 5 test datasets and standard deviation), and the features ranked most important (out of all features reported) for the best models. Specificity, sensitivity, positive and negative prediction values were also reported for the best model and the stability of important features across folds (using Spearman correlation).

Figures were created using the packages ggplot2 (v3.5.1)^64^ and pROC (v1.18.5)^65^, and descriptive tables using table1 (v1.4.3)^66^ in R.

## Supplementary information

Supplementary Information

## Data Availability

Microbiome data and limited metadata are available in the European Nucleotide Archive (ENA) under project name PRJEB81814. Parts of the code are available at https://github.com/ctmrbio/ML_swemami. Other unpublished codes may be made available to qualified researchers at the corresponding author's reasonable request.

## Code availability

Parts of the code are available at https://github.com/ctmrbio/ML_swemami. Other unpublished codes may be made available to qualified researchers at the corresponding author’s reasonable request.
