# Supplementary material for: Machine learning and the role of the vaginal and fecal microbiome in miscarriage: a matched case-control study
Source: NPJ Biofilms Microbiomes. 2026 Mar 13;12:66. doi: 10.1038/s41522-026-00956-2 (PMC13009249; doi:10.1038/s41522-026-00956-2)
Supplement: Supplementary file 1 — Supplementary Information [file 41522_2026_956_MOESM1_ESM.pdf]

Supplementary Materials

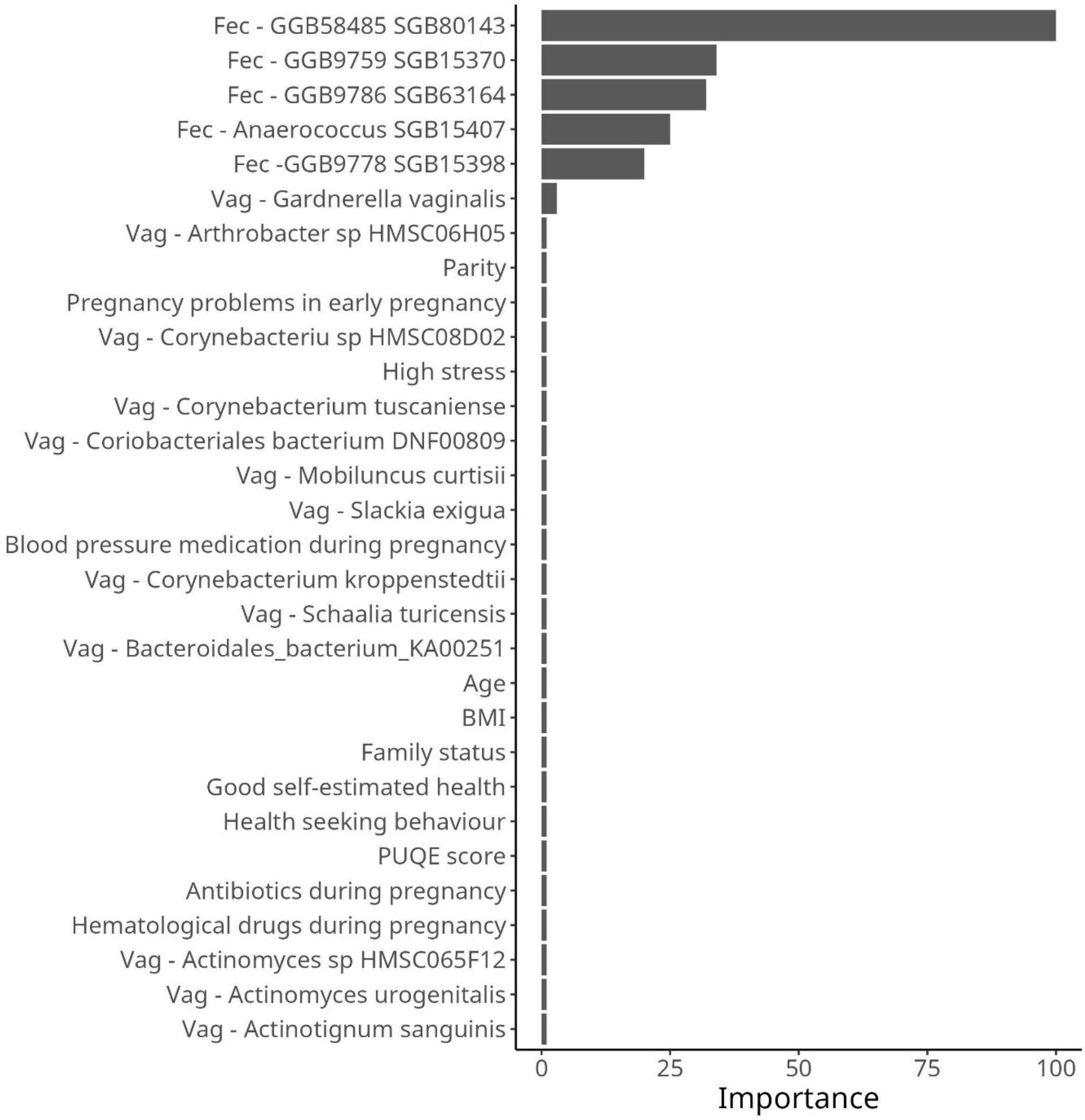

Figure S1: Importance of the top 30 variables included in the Ranger Random Forest model

Table S1: Univariable and multivariable logistic regression assessing risk factors for miscarriage using a questionnaire, and fecal and vaginal microbiome data. Results are shown as p values and adjusted odds ratios (aOR) with 95% confidence intervals (CIs). Variables with p<0.2 (in bold) in univariable logistic regression were included in the multivariable model, as well as age, BMI, pregnancy week of sampling and pre/post-COVID-19 start in Sweden (defined as March 1st, 2020).

		**Univariable**	**Multivariable**	
		**p-value**	**aOR, 95%CI**	**p-value**
**Background factors**				
Age				
	< 25	0,76		
	25-35	REF		
	> 35	0,49		
BMI prior to pregnancy				
	< 18.5 Underweight	0,52		
	18.5-25 Normal weight	REF		
	> 25 Overweight	0,97		
Swedish born		0,27		
University education		0,88		
Family status		NA		
Socioeconomic score		0,71		
**Gynecological health**				
Ever had HPV***		0,39		
Ever had dysplasia		0,7		
HPV vaccinated		0,91		
VALENCIA† group				
	CST-I	REF	REF	REF
	CST-II	**0,06**	**6.52 (1.58-26.98)**	**0,01**
	CST-III	0,81	1.20 (0.31-4.65)	0,8
	CST-IVB	0,25	**4.18 (1.08-16.18)**	**0,04**
	CST-IVC	0,32	9.20 (0.48-177.8)	0,14
	CST-V	0,99	NA	NA
HPV				
	Any HPV	**0,19**		
	High risk HPV	0,92		
	Low risk HPV	0,4		
	Vaccine type HPV	0,99		
	Non-vaccine type HPV	**0,09**	**7.74 (1.67-35.89)**	**0,01**
Vaginal microbiome				
	Shannon index	0,75		
	Inverse Simpson	0,59		
	Pielou’s evenness	0,7		
	Richness	0,85		

Fecal microbiome				
	Shannon index	0,37		
	Inverse Simpson	**0,09**	0.99 (0.96-1.03)	0,7
	Pielou's evenness	0,11		
	Richness	0,66		
**General health**				
Smoking		NA		
Snuff / smokeless tobacco		NA		
Good self-health estimation		0,85		
Health seeking behavior		NA		
Daily fiber		0,26		
Eating disorder		0,55		
**Pregnancy characteristics**				
Primiparous		0,35		
Alcohol during pregnancy		0,66		
Regular menstruation		**0,14**	**0.16 (0.03-0.78)**	**0,02**
High stress early pregnancy		0,49		
EPDS* depression score early pregnancy		0,99		
Natural conception		0,48		
Time to pregnancy				
	0-6	REF		
	6-12	0,52		
	> 12	0,28		
Any pregnancy problems		0,77		
PUQE** score				
	Mild	REF		
	Moderate	0,24		
	Severe	0,77		
Bristol stool scale rank				
	Fast transit	0,32		
	Normal transit	REF		
	Slow transit	0,29		
	Various transit	0,94		
**Drug use during pregnancy**				
Any drugs		0,71		
Multiple drugs		0,4		
Allergy or antihistamine medication		0,63		
Neurological medication		0,48		
Hematological medication		0,39		
Blood pressure medication		0,99		
Antibiotics		0,57		

Table S2: Results from univariable PERMANOVA analysis on questionnaire variables the vaginal and fecal microbiome of miscarriage cases and matched controls. P< 0.05 are marked in bold, and R2 values are reported for significant variables.

**Questionnaire variable**	**Vaginal microbiome**		**Fecal microbiome**	
	P value	R^2	P value	R^2
Age	0,51		**0,03**	0,02
BMI	0,29		0,3	
Swedish born	0,58		**0,04**	0,01
Education level	0,94		**0,05**	0,01
Family status	0,21		0,74	
SES score	0,64		0,13	
Parity	0,09		**0,002**	0,01
Alcohol during pregnancy	0,6		0,95	
Self-estimated health	0,23		0,66	
Health seeking behavior	0,95		0,86	
Daily fiber	0,92		0,19	
Diagnosed eating disorder	0,51		0,8	
Regular menstruation	0,15		0,87	
High stress	0,5		0,17	
Depression score	0,48		0,37	
Natural conception	0,11		0,53	
Time to pregnancy	0,16		0,39	
Pregnancy problems	0,56		0,63	
PUQE score	0,33		0,19	
Bristol stool scale	0,47		0,26	
Any drugs	0,81		0,62	
Multiple drugs	0,75		0,59	
Allergy or antihistamines	0,41		0,37	
Neurological medication	0,56		0,46	
Hematological medication	0,39		**0,05**	0,01
Blood pressure medication	0,08		**0,05**	0,01
Antibiotics	0,05		0,19	
Ever HPV positive	0,63		0,19	
Ever dysplasia	0,37		0,67	
HPV vaccination	0,97		0,2	
Any HPV	0,72		-	
High-risk HPV	0,07		-	
Low-risk HPV	**0,02**	0,03	-	
Vaccine-type HPV	**0,002**	0,05	-	
Non-vaccine-type HPV	0,54		-	

Table S3: Questionnaire variables from early pregnancy included in the logistic regression analysis and machine learning models.

**Background variables**	**Categories**
Age	<25, 25-35, >35
BMI* before pregnancy	Underweight (<18.5), Normal weight (18.5-25), Overweight and obese (>25)
Swedish born	Yes/No
University education	Yes/No
Family status	In a relationship, single
Socioeconomic score	Low, high
**Gynecological health**	
Ever had HPV**	Yes/No
Ever had dysplasia	
HPV vaccination	
**General health**	
Smoking	Yes/No
Snuff / smokeless tobacco	
Good self-health estimation	
Health seeking behavior	
Daily fiber	
Eating disorder	
Prior mental diseases	
**Pregnancy characteristics**	
Primiparous	Yes/No
Alcohol during pregnancy	Yes/No
Regular menstruation	Yes/No
High stress early pregnancy	Highest quartile of PSS-4 (>6)
EPDS‡ score	Continuous (0-X
Natural conception	Yes/No
Time to pregnancy	0-6 months, 6-12 months, >12 months
Any pregnancy problems	Yes/No
PUQE†† score	Mild, moderate, severe
Bristol stool scale early pregnancy	Fast transit, Normal transit, Slow transit, Various
**Drug use during pregnancy**	
Any drugs	Yes/No
Multiple drugs	
Allergy or antihistamine medication	

Neurological medication		
Hematological medication		
Blood pressure medication		
Antibiotics		
*Body mass index, **Human Papillomavirus, ‡Edinburgh Postnatal Depression Scale,		
††Pregnancy-Unique Quantification of Emesis score		

Table S4: AUROC values for each of the four datasets, over the five testing cohorts, for the six machine learning algorithms for both full model and feature selection.

Data: All combined		Full model							Feature selection							
	Test set	svm	enet	ranger	rf	knn		**30**	**20**	**10**	**5**					
	1	61	51	77	73	62		73	72	73	75					
	2	59	62	72	70	62		70	69	72	70					
	3	72	70	89	80	67		89	88	86	84					
	4	67	66	86	81	63		84	84	84	86					
	5	59	70	88	84	56		86	84	84	88					
	Mean	**64**	**64**	**82**	**78**	**62**		**80**	**79**	**80**	**81**					
	SD	6	8	8	6	4		8	8	7	8					
	Accuracy	58	58	73	68	58		70			74					
Data: Fecal microbiome																
		svm	enet	ranger	rf	knn	nnet		**20**	**10**	**5**					
	1	47	50	80	77	55	62		73	73	75					
	2	48	69	72	69	57	52		73	69	70					
	3	73	89	81	80	66	55		86	88	84					
	4	64	77	83	80	52	64		88	84	86					
	5	59	78	80	76	50	63		86	88	89					
	Mean	**58**	**73**	**79**	**76**	**56**	**59**		**81**	**80**	**81**					
	SD	11	15	4	5	6	5		8	9	8					
	Accuracy	43	54	74	76	60	59				74					
Data: Vaginal microbiome																
		svm	enet	ranger	rf	knn	nnet	**80**	**70**	**60**	**50**	**40**	**30**	**20**	**10**	**5**
	1	84	56	73	48	50	62	75	80	66	64	64	64	52	52	52
	2	83	66	69	59	56	59	67	77	75	67	62	61	59	45	59

	3	84	84	83	62	56	89	73	83	67	72	70	72	44	42	44
	4	88	80	84	64	50	80	67	78	69	62	70	64	50	45	50
	5	86	64	77	69	56	64	59	70	72	56	59	58	34	42	34
	Mean	**85**	**70**	**77**	**60**	**54**	**71**	**68**	**78**	**70**	**64**	**65**	**64**	**48**	**45**	**48**
	SD	2	12	6	8	3	13		5	4	6	5	5	9	4	9
	Accuracy	30	55	55	64	54	54									
Data: Questionnaire																
		svm	enet	ranger	rf	knn			**20**	**10**	**5**					
	1	77	58	73	77	40			80	62	69					
	2	70	55	64	67	30			77	47	40					
	3	84	56	80	80	61			88	61	77					
	4	84	52	67	59	59			88	45	59					
	5	69	56	75	69	45			75	61	65					
	Mean	**77**	**55**	**72**	**70**	**47**			**82**	**55**	**62**					
	SD	7	2	6	8	13			6	8	14					
	Accuracy	45	63	59	60	50			81							
